# Supplementary figures and images for: The claudin-like apicomplexan microneme protein is required for gliding motility and infectivity of Plasmodium sporozoites
Source: PLoS Pathog. 2023 Mar 16;19(3):e1011261. doi: 10.1371/journal.ppat.1011261 (PMC10047546; doi:10.1371/journal.ppat.1011261)

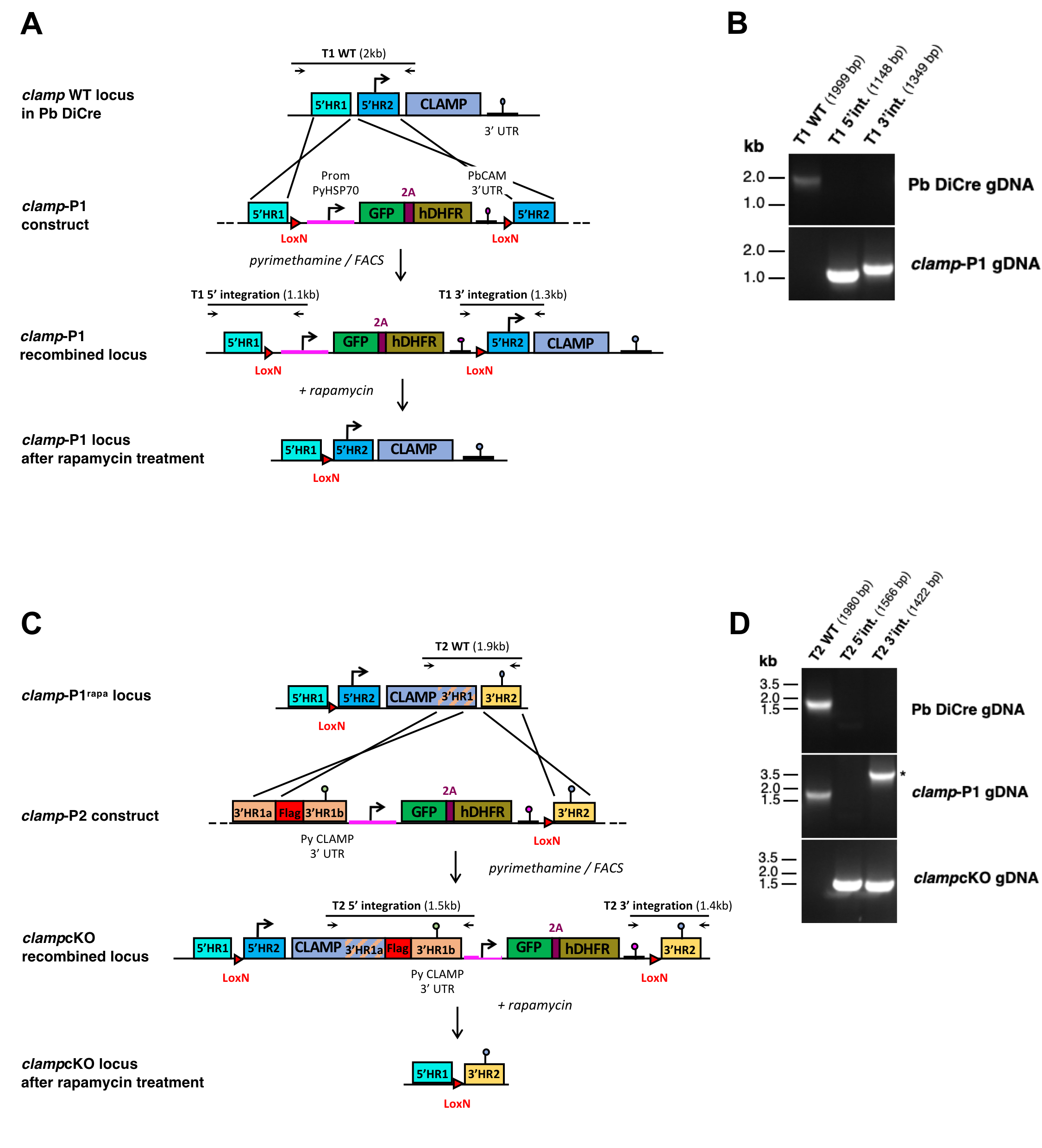

Supplement: S1 Fig — A. Detailed strategy to insert a LoxN site upstream of clamp gene using the P1 construct. Upstream homology regions (5’HR1 and 5’HR2) were inserted in the pUpstream2Lox plasmid on each side of a GFP-2A-hDHFR cassette flanked by two LoxN sites. The P1 construct was transfected into mCherry-expressing PbDiCre parasites. Following parasite transfection and selection with pyrimethamine, mCherry+/GFP+ parasites were sorted by flow cytometry to exclude any residual GFP- population. Rapamycin-induced excision lead to removal of the GFP-2A-hDHFR cassette and the retention of a single LoxN site upstream of clamp. Genotyping primers and expected PCR fragments are indicated by arrows and lines, respectively. B. PCR analysis of genomic DNA isolated from parental PbDiCre and clamp-P1 parasites. Confirmation of the predicted recombination events was assessed with primer combinations specific for WT, 5’ or 3’ integration for the first transfection (T1). Primers used for genotyping are listed in S2 Table. C. Detailed strategy to insert a LoxN site downstream of clamp gene using the P2 construct. Downstream 3’ homology regions (3’HR1 and 3’HR2) were inserted in the pDownstream1Lox plasmid on each side of a GFP-2A-hDHFR cassette, flanked on one side by a single LoxN site. A triple Flag epitope tag (3xFlag) was inserted in frame with clamp ORF immediately before the STOP codon. In addition, a 559 bp fragment corresponding to the 3’ UTR sequence from P. yoelii clamp gene was inserted immediately downstream of STOP codon, to allow proper gene expression and avoid spontaneous recombination with the 3’ UTR of P. berghei clamp, which was used as 3’HR2. The P2 construct was transfected into rapamycin-treated mCherry+/GFP- clamp-P1 parasites (clamp-P1rapa). Following parasite transfection and selection with pyrimethamine, mCherry+/GFP+ parasites were sorted by flow cytometry to exclude any residual GFP- population, and cloned by limiting dilution and injection into mice, resulting in the fin [file ppat.1011261.s004.tif]

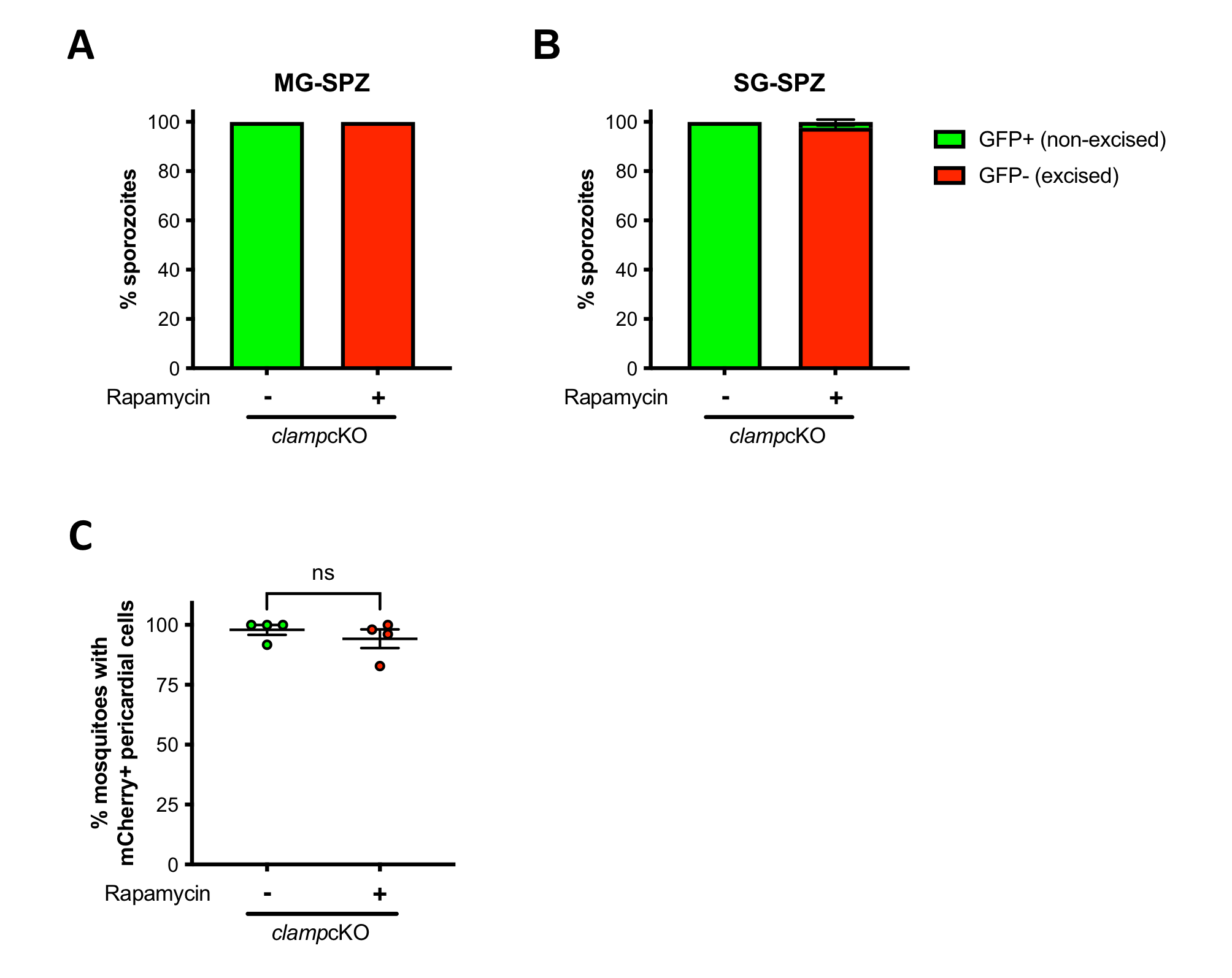

Supplement: S2 Fig — A-B. Fluorescence-based quantification of excised (mCherry+GFP-) and non-excised (mCherry+GFP+) sporozoites collected from midguts (A) and salivary glands (B) of female mosquitoes infected with rapamycin-exposed and untreated clampcKO parasites. Results shown are based on observation of at least 200 sporozoites per condition and per experiment (mean +/- SEM of four independent experiments). C. Quantification of infected female mosquitoes exhibiting mCherry-labelled pericardial cells 16 days post-infection, based on observation of at least 50 mosquitoes per condition (mean +/- SEM of four independent experiments). Ns, non-significant (Two-tailed ratio paired t test). (TIF) [file ppat.1011261.s005.tif]

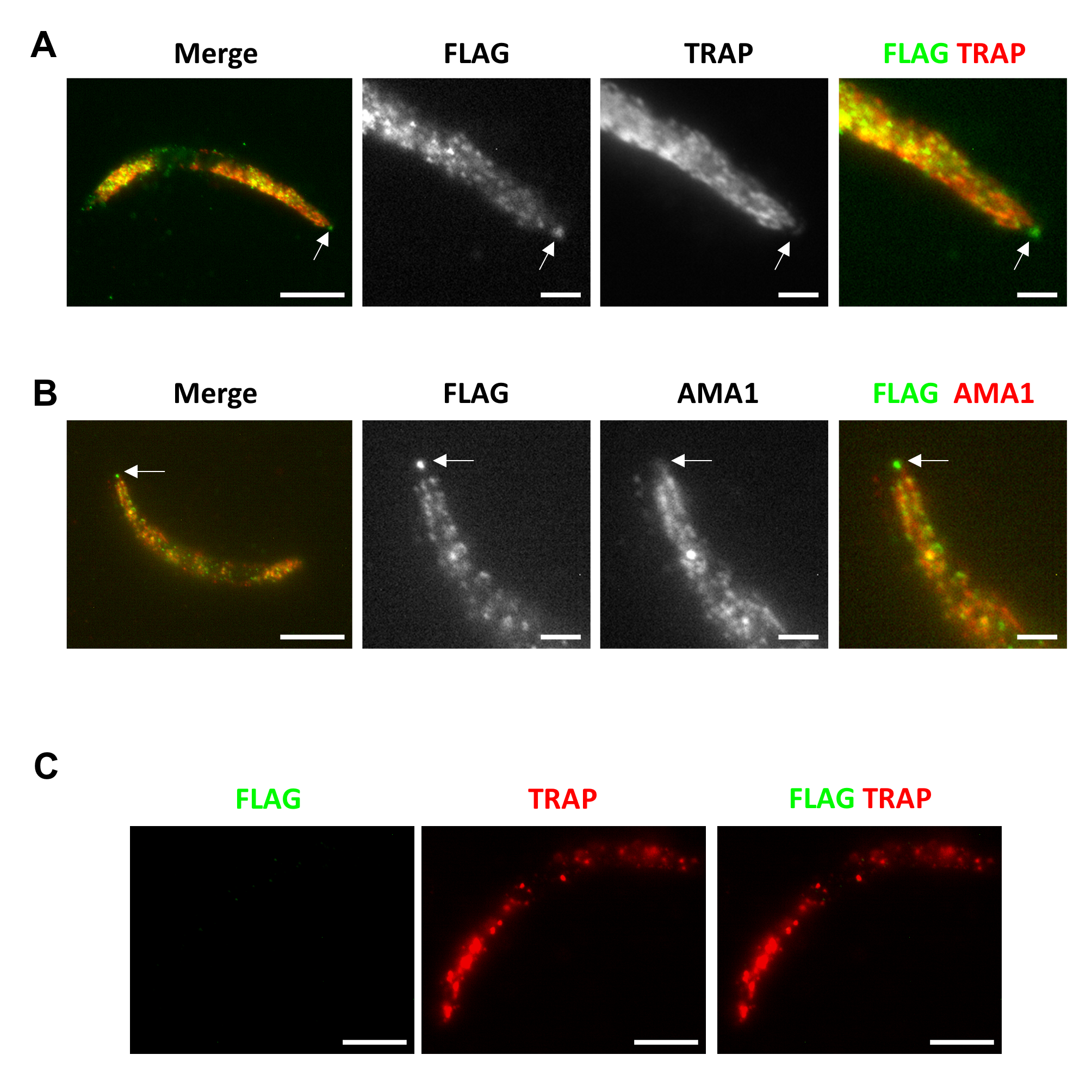

Supplement: S3 Fig — A-B. Salivary gland sporozoites expressing Flag-tagged CLAMP (untreated clampcKO parasites) were examined by expansion microscopy using antibodies against Flag (green) and TRAP (in A, red) or AMA1 (in B, red). Specific accumulation of CLAMP is clearly visible at the apical tip of sporozoites (arrows). Scale bars, 10 μm. C. Rapamycin-treated clampcKO parasites were examined by expansion microscopy after labeling with antibodies against Flag (green) and TRAP (red). The absence of signal with anti-Flag antibodies confirms the efficient depletion of CLAMP following rapamycin-induced gene excision. Scale bars, 10 μm. (TIF) [file ppat.1011261.s006.tif]

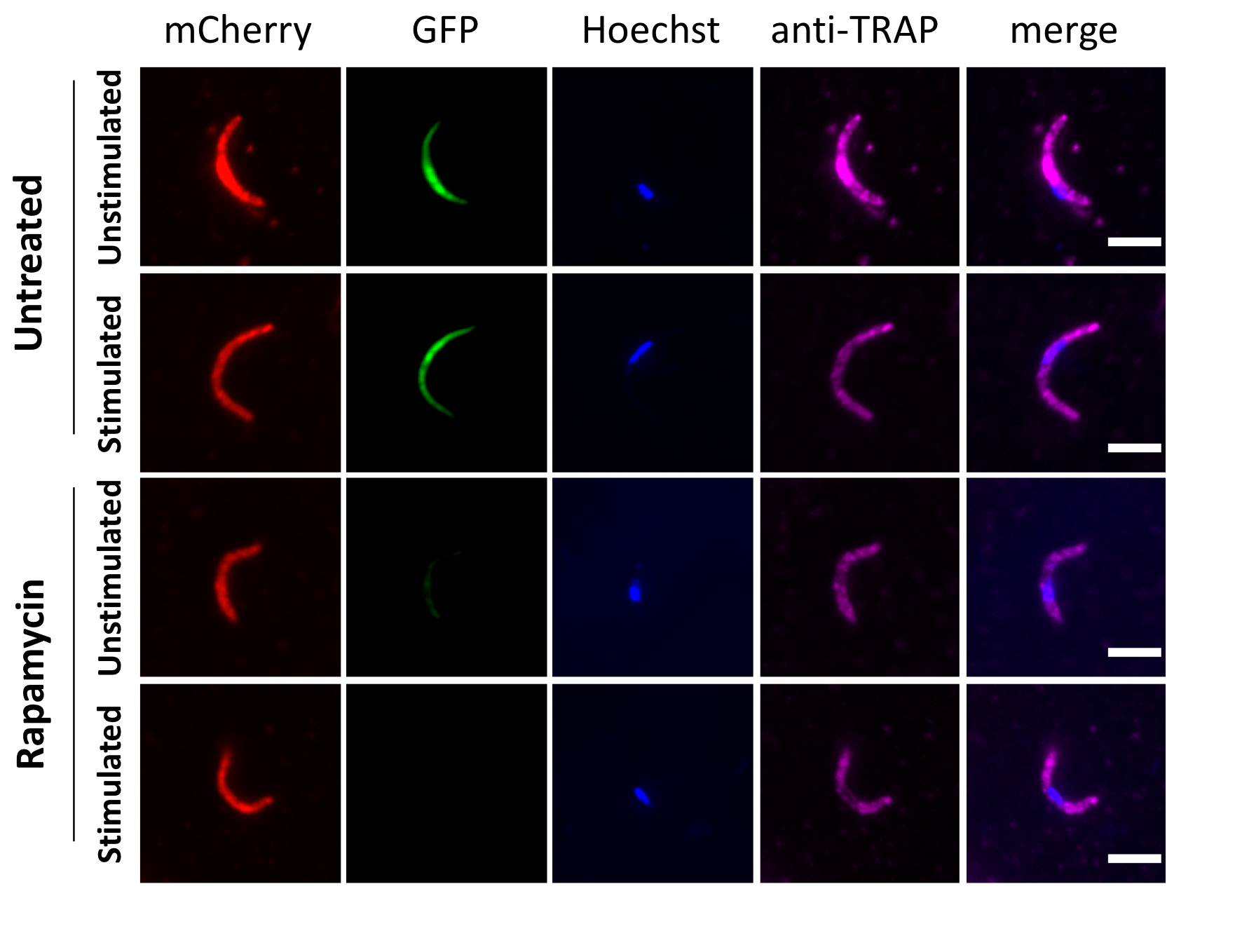

Supplement: S4 Fig — Sporozoites were collected from the salivary glands of mosquitoes infected with untreated or rapamycin-exposed clampcKO parasites. Microneme secretion was stimulated by incubation at 37°C in the presence of 1% BSA and 1% ethanol for 15 min. Stimulated and unstimulated sporozoites were then fixed with 4% PFA without permeabilization, and stained with anti-TRAP antibodies (magenta) and the nuclear stain Hoechst 33342 (blue). Untreated parasites express GFP (green) and mCherry (red), while rapamycin-treated parasites express mCherry only. Scale bars, 5 μm. (TIF) [file ppat.1011261.s007.tif]
