# Supplementary material for: The claudin-like apicomplexan microneme protein is required for gliding motility and infectivity of Plasmodium sporozoites
Source: PLoS Pathog. 2023 Mar 16;19(3):e1011261. doi: 10.1371/journal.ppat.1011261 (PMC10047546; doi:10.1371/journal.ppat.1011261)
Supplement: Attachment — Submitted filename: Loubens_Response to reviewers.pdf [file ppat.1011261.s009.pdf]

Point-by-point response PPATHOGENS-D-22-01506 (our responses in blue)

Editor

As you will see, the reviewers agreed that the work is of very high quality and that it generates important data regarding the function of CLAMP in sporozoite biology, as well as demonstrating the applicability of the DiCre conditional gene disruption system in insect stages of Plasmodium. However, Reviewer #1 believes that the work produces few new mechanistic insights into the functional interactions between CLAMP and its partner proteins, and specifically how CLAMP modulates TRAP function. The Editorial Board fully concurs with this view, especially in the context of previous studies on CLAMP. The apparent physical interaction with TRAP and the putative pantothenate transporter PAT (but please see PMID: 34969059 regarding PAT) is intriguing, but to provide the level of new insight to warrant further consideration by PLoS Pathogens it is essential that the authors better elucidate the mechanism(s) underlying the apparent regulation of TRAP secretion by CLAMP. Accordingly, we would be willing to consider a revised form of the manuscript if you can provide such data. As suggested by the Reviewer, this could comprise more detailed analysis of the interaction between the proteins that co-precipitate in your experiments (do they form a multimolecular complex?), as well as information on the subcellular localisation of the partner proteins in sporozoites.

We would like to thank the editor and the reviewers for their positive and useful comments. As detailed below in our point-by-point response, we have now addressed all the comments. In particular, we included co-IP and immunoblotting results to validate the mass spectrometry data showing an interaction between CLAMP and TRAP. We don't have parasites with dual genetic modifications (for example CLAMP tagged and TRAP or PAT knockout). Generating such mutants is technically challenging. Therefore we cannot perform the suggested experiments to ascertain whether the three proteins interact directly and form a tripartite complex. We cannot ascertain whether CLAMP directly interacts with TRAP, since in our co-IP experiments interactions could be indirect via a common partner. Nevertheless, the cognate mutants share a common phenotype, consistent with a functional link between these proteins.

We thank the editor for pointing at the potential functions of PAT. In *Toxoplasma*, the homolog of PAT is a protein called TFP1, which is also involved in microneme exocytosis but acts upstream of secretion during organelle maturation (PMID 29738095). We have added this point in the revised manuscript.

Importantly, we have performed expansion microscopy experiments and included the data in the revised manuscript. The results show colocalization of CLAMP and TRAP in a subset of micronemes, and reveal a unique accumulation of CLAMP at the apical tip of sporozoites. This apical localization is reminiscent of proteins recently described in *Toxoplasma* that control rhoptry discharge. These results raise the possibility that CLAMP has a dual function in sporozoites, in controlling TRAP-mediated gliding motility and rhoptry discharge for host cell invasion. Such a model could reconcile the distinct phenotypes observed between CLAMP mutants in *Toxoplasma* (work from the Lourido lab) and *P. berghei* (this study).

While we acknowledge that future studies will be necessary to elucidate the molecular function of the protein in Apicomplexa, we believe that our study provides novel and important insights into the function of CLAMP in *Plasmodium* sporozoites.

Major Issues: Key Experiments Required for Acceptance

Reviewer #1: More mechanistic insight is needed as to the connection between CLAMP, TRAP and the pantothenate transporter. Are those proteins localizing in the micronemes together? This could be addressed with immuno electron microscopy and/or by performing pulldowns in parasite lines lacking either TRAP or panthothenate transporter.

To address this comment we performed a series of additional experiments and included new data in the revised manuscript.

We performed a new co-IP experiment that was analyzed by immunoblotting and which confirmed the interaction between CLAMP and TRAP (**new Figure 5B**), validating the mass spectrometry data. We cannot ascertain whether CLAMP directly interacts with TRAP, as in the co-IP experiments interactions could be indirect, for example via a common partner. Nevertheless, the cognate mutants share a common phenotype, consistent with a functional link between these proteins. Unfortunately, we do not have parasites expressing a tagged version of CLAMP and lacking TRAP or PAT, so we could not perform the suggested experiment to ascertain whether the three proteins are interdependent and form a tripartite complex. Please note that generating such mutants with dual genetic modifications (for example CLAMP tagged and TRAP or PAT knockout) is technically challenging. However we plan to explore further interactions between CLAMP and micronemal proteins, including using genetic tools. We hope that the reviewer will agree that such follow up work falls out of the scope of the present study.

Most importantly, we analyzed in details the localization of CLAMP and TRAP by applying for the first time expansion microscopy (U-ExM) with *Plasmodium* sporozoites. U-ExM allowed visualizing individual micronemes in sporozoites, and revealed partial codistribution of CLAMP and TRAP in a subset of micronemes (**new Figure 6A** and **S3**). In contrast, CLAMP and AMA1 were not colocalized (**new Figure 6B**), supporting the existence of distinct microneme populations in sporozoites. We also attempted to visualize CLAMP subcellular localization by immune-EM, but failed to detect the CLAMP-Flag protein. We suspect a problem of detection of the Flag epitope with our anti-Flag antibodies as we had the same issue with another (unrelated) Flag-tagged parasite.

Collectively, we believe that our data strongly support a functional role of CLAMP in TRAP-mediated gliding motility: 1) CLAMP, like TRAP, is required for gliding motility, as shown by conditional mutagenesis; 2) CLAMP and TRAP colocalize in a subset of micronemes, as shown by U-ExM; 3) CLAMP and TRAP interact, as shown by co-IP; and 4) CLAMP is required for TRAP shedding. Future work will be required to unravel how CLAMP controls TRAP secretion, and whether CLAMP specifically regulates TRAP or more generally microneme secretion.

Remarkably, U-ExM also revealed a unique accumulation of CLAMP at the apical tip of sporozoites, which was not observed with TRAP or AMA1 (**new Figure 6**). This apical

localization is reminiscent of proteins recently described in *Toxoplasma* that control rhoptry discharge (Nd6 and CRMPs). These results raise the possibility that CLAMP has a dual function in sporozoites, in controlling TRAP-mediated gliding motility on one hand, and rhoptry discharge for host cell invasion on the other. Such a model would reconcile the distinct phenotypes observed between CLAMP mutants in *Toxoplasma* (work from the Lourido lab) and *P. berghei* (this study). In this regard, a recent paper from the Lourido lab (BioRXiv, https://doi.org/10.1101/2022.11.28.518173) shows that CLAMP forms a tripartite complex with SPATR and a protein called CLIP in *Toxoplasma gondii*. Interestingly, SPATR is dispensable in *Toxoplasma* and is not required for gliding motility in this parasite, in contrast with its essential role in *Plasmodium* blood stages and during gliding motility in sporozoites. These observations mirror our functional data with CLAMP conditional knockout in *P. berghei*, and strengthen the hypothesis of functional divergence between *Plasmodium* and *Toxoplasma*.

We have extensively modified the discussion section in our revised manuscript to take into account:

1) elements supporting the functional link between CLAMP and TRAP-mediated gliding motility (lines 418-422 in the clean version);
2) our new U-ExM results, especially the apical localization (lines 471-478);
3) the *Toxoplasma* CLAMP complex data from the Lourido lab (lines 457-470);
4) possible models of action of CLAMP in sporozoites (lines 422-432 and 478-486).

We hope that the reviewer will be convinced that our study provides novel and important insights into the function of CLAMP in *Plasmodium* sporozoites, although future studies will be necessary to elucidate the molecular function of the protein, not only in *Plasmodium* sporozoites but more generally in Apicomplexa.

Minor Issues: Editorial and Data Presentation Modifications

Reviewer #1: Likely three of the six proteins found in the pulldown or contaminants (ELF, actin, tubulin) and this should be stated more clearly.

We agree with the reviewer that eEF1alpha and tubulin are likely contaminants in the pulldown experiments. In contrast, it is not necessarily the case for actin, considering the link between CLAMP, TRAP and motility. We have added this point in the revised text (lines 300-302 in the clean version).

I would suggests to review the way the figures are arranged as follows. Figure 1: the panel C could be placed next to panel A and panel B could be enlarged.

We have modified Fig 1 following the reviewer's suggestions.

Figure 3: maybe the Höchst and merge signals are not needed. Could the images be enlarged and the micronemes pointed at?

Fig 3 was intended to confirm depletion of CLAMP in rapamycin-exposed parasites. Therefore it was left unchanged in the revised manuscript. However, we now provide new data on the localization of CLAMP in micronemes using U-ExM, in the **new Figures 6** and **S3**.

Figure 4: the legend to panel C could be moved between the two circles to align the panels.

We modified the figure as suggested by the reviewer.

Reviewer #2: Figure 2C: I agree with the authors, that the major defect from clamp deletion is seen in the number of sporozoites in the mosquito salivary glands. However, despite failing to invade the salivary glands, there is no accumulation of clampcKO sporozoites in the hemolymph. Could this be explained by the difference seen when comparing the numbers of midgut sporozoites in clampcKO-infected mosquitoes with the non-treated parasites?

We cannot exclude that the lack of accumulation of sporozoites in the haemolymph is due in part to the slight reduction in midgut sporozoite numbers (as shown in Fig 2C). However, a more likely explanation is that free sporozoites are rapidly eliminated from the mosquito circulation. This is corroborated by the fluorescence of pericardial cells in the mosquito abdomen, reflecting uptake of egressed parasites. Similar observations were made with AMA1, RON2 and RON4-deficient sporozoites, which like CLAMP mutants fail to invade the mosquito salivary glands, but unlike CLAMP cKO show no defect in gliding motility (PMID 35731833). We have added a comment in the revised text addressing the reviewer's question (lines 191-193 in the clean version).

Figure 4A and B: Sporozoites used in these assays are from salivary glands? In the text is written this is the case for the gliding motility assays (Fig4C) but not clear for cell traversal and infection experiments. From the numbers used in the assay and the results it seems so but I suggest clarifying.

The experiments shown in Fig 4a, 4B and 4C were all performed with salivary gland sporozoites. This is now explicitly indicated in the figure legend.

Figure 1C: Please confirm if only one experiment was performed.

The reviewer is correct, the experiment in Fig 1C was performed only once, as it was designed essentially to verify the efficiency of the conditional knockout and the essentiality of CLAMP in asexual blood stages. This is now specified in the figure legend.

Line 176: Since no additional purification step is performed, I suggest the use of the term "sporozoites were collected" and not "isolated". Check all the manuscript.

As suggested, we have modified the text throughout the manuscript.

Line 575: Please include the detailed composition of the lysis buffer as this may affect the lysate composition on membrane-bound proteins.

We now provide the detailed composition of the lysis buffer in the methods section (10mM Tris pH 7.5, 150mM NaCl, 0.5 mM EDTA, 0.5% w/v NP40 and protease inhibitors) (line 637 in the clean version). Under these conditions, TRAP was co-immunoprecipitated with CLAMP as shown by mass spectrometry (Fig 5A and Table S1) and western blot (**new Fig 5B**).
